# Lipidomic profiling of the developing kernel clarifies the lipid metabolism of *Paeonia ostii*

**DOI:** 10.1038/s41598-021-91984-9

**Published:** 2021-06-15

**Authors:** Shui-Yan Yu, Ying Zhang, Yu-Ping Lyu, Zu-Jie Yao, Yong-Hong Hu

**Affiliations:** Shanghai Key Laboratory of Plant Functional Genomics and Resources, Shanghai Chenshan Botanical Garden, Shanghai, 201602 China

**Keywords:** Lipidomics, Lipids

## Abstract

Lipid components in the developing kernel of *Paeonia ostii* were determined, and the fatty acid (FA) distributions in triacylglycerol and phospholipids were characterized. The lipids in the kernel were mainly phospholipids (43%), neutral glycerides (24%), fatty acyls (26%), and sphingolipids (4.5%). The dominant neutral glycerides were TAG and diacylglycerol. The PL components included phosphatidic acid, phosphatidyl glycerol, phosphatidyl choline, phosphatidyl serine, phosphatidyl inositol, and phosphatidyl ethanolamine. As the kernel developed, the profiles of the molecular species comprising TAG and PL changed, especially during the earlier phases of oil accumulation. During rapid oil accumulation, the abundances of sphingosine-1-phosphate, pyruvic acid, stearic acid, and alpha-linolenic acid changed significantly; the sphingolipid metabolism and unsaturated FAs biosynthesis pathways were significantly enriched in these differentially abundant metabolites. Our results improve our understanding of lipid accumulation in tree peony seeds, and provide a framework for the analysis of lipid metabolisms in other oil crops.

## Introduction

The tree peony (*Paeonia* section *Moutan* DC.), a historically important royal flower, has been cultivated in China for nearly 2000 years^1^. Nine wild species have been described in section *Moutan*, and all are native to China^2^. Tree peonies, which are woody, oleaginous plants, have become increasingly commonly cultivated due to the high-quality oil that is produced in their seeds; this oil has attracted significant economic and academic interest. Tree peony oil is characterized by high levels of unsaturated fatty acids (UFAs; > 90%), including alpha linolenic acid (ALA; > 40%), linoleic acid (LA), and oleic acid (OA)^3,4^. The seeds of *Paeonia ostii* and *P. rockii* were identified as a novel source of high-quality edible oils in 2011 by the National Health Commission of the People’s Republic of China^5^. In particular, *P. ostii* is a prospective industrial crop, as ALA levels in *P. ostii* seeds are high, and this plant is already widely cultivated in many parts of China^6^.

Previous studies of peony seed oil have mainly focused on the types and contents of FAs in tree peonies, including wild species and cultivated varieties of a variety of provenances; other studies have investigated the influence of paternal inheritance on FA content^3,4,6–8^. The synthetic process that leads to the accumulation of triacylglycerol (TAG), in seed oil bodies begins with the de novo synthesis of FAs in plastids^9–12^. The synthesized FAs are exported to the endoplasmic reticulum (ER) for acyl editing or to the plastid for desaturation^13^. Modified FAs are then assembled into TAG via the Kennedy pathway in the ER, dependent on the acetyl CoA pool^14^, or via the phospholipid:diacylglycerol acyltransferase (PDAT) pathway, independent of the acetyl CoA pool^15^. The ALA biosynthesis and TAG assembly pathways in the tree peony have been investigated using transcriptomics, proteomics, and microRNA^16–20^. Several such studies have focused on the cloning, expression, and function of key genes in the ALA metabolism of *P. suffruticosa* (including wild species and cultivated varieties), including stearoyl-ACP desaturase (SAD), fatty acid desaturase 2 (FAD2), FAD3, FAD8, lysophosphatidic acid acyltransferase (LPAAT), diacylglycerol acyltransferase, (DGAT), PDAT, and oleosin^19,21–24^.

Polyunsaturated fatty acids (PUFAs) like ALA are stored in seeds as oil droplets; this storage mechanism involves many complex metabolic pathways, which require various lipid molecules as ligands or donors, as well as other lipid molecules as signal factors to regulate metabolic pathways^25^. Although studies of FA biosynthesis in the tree peony have explored FA-associated metabolic pathways and gene function, little is known about the intracellular lipid species. In oil seeds, the formation of ALA TAG from LA under the action of delta-15 FA desaturase is only the most direct step in the process of ALA synthesis in cells; this process also involves acyl editing, the Lands cycle, and TAG assembly^13^. Thus, differences in the relative abundances of lipid species, such as phosphatidylcholine (PC), lysophosphatidylcholine (LPC), phosphatidylethanolamine (PE), diacylglycerol (DAG), and TAG, among oil crops is of substantial importance.

To the best of our knowledge, no previous studies have characterized the lipid molecular species in the *P. ostii* kernel. To address this knowledge gap in the present study, we identified the lipid species in the developing kernel and investigated the distributions of FAs in the TAGs and major phospholipids (PLs). The results of this study will provide a basis from which to elucidate further details of the lipid metabolism and will also improve our understanding of mechanisms regulating tree-peony oil metabolism.

## Results and discussion

### Total lipid content during *P. ostii* seed kernel development

At the study location (south of the Yangtze River), *P. ostii* seed development lasted about 120 days. During the early stage of seed development (7–35 DAF), total lipid accumulation was limited (5.71% at 35 DAF), but by the end of kernel cell stage (35–42 DAF), total lipid content had increased (7.66% at 42 DAF). Between 42 and 49 DAF, total lipid content increased significantly to 11.84%, nearly double the accumulation at 35 DAF (Supplementary Fig. S1). During cotyledon embryo formation (70–77 DAF), total lipid content increased to 38.62%, nearly 8 times greater than that at 35 DAF. Between the end of seed development (91 DAF) and maturity (119 DAF), total lipid content decreased slightly, from 38.26% to 29.99%. Total lipid content peaked during cotyledon embryo formation, exhibiting a bell-shaped curve throughout the seed development process. Four samples, corresponding with significant changes in lipid accumulation during seed-kernel development, were selected for further lipidomic analysis: minimal accumulation (35 DAF; sample set T1), initial accumulation (49 DAF; sample set T2), peak accumulation (77 DAF; sample set T3), and maturity (119 DAF; sample set T4).

### UPLC-Q-TOF–MS/MS analysis of the lipid extracts from *P. ostii* seeds

**Figure 1:**
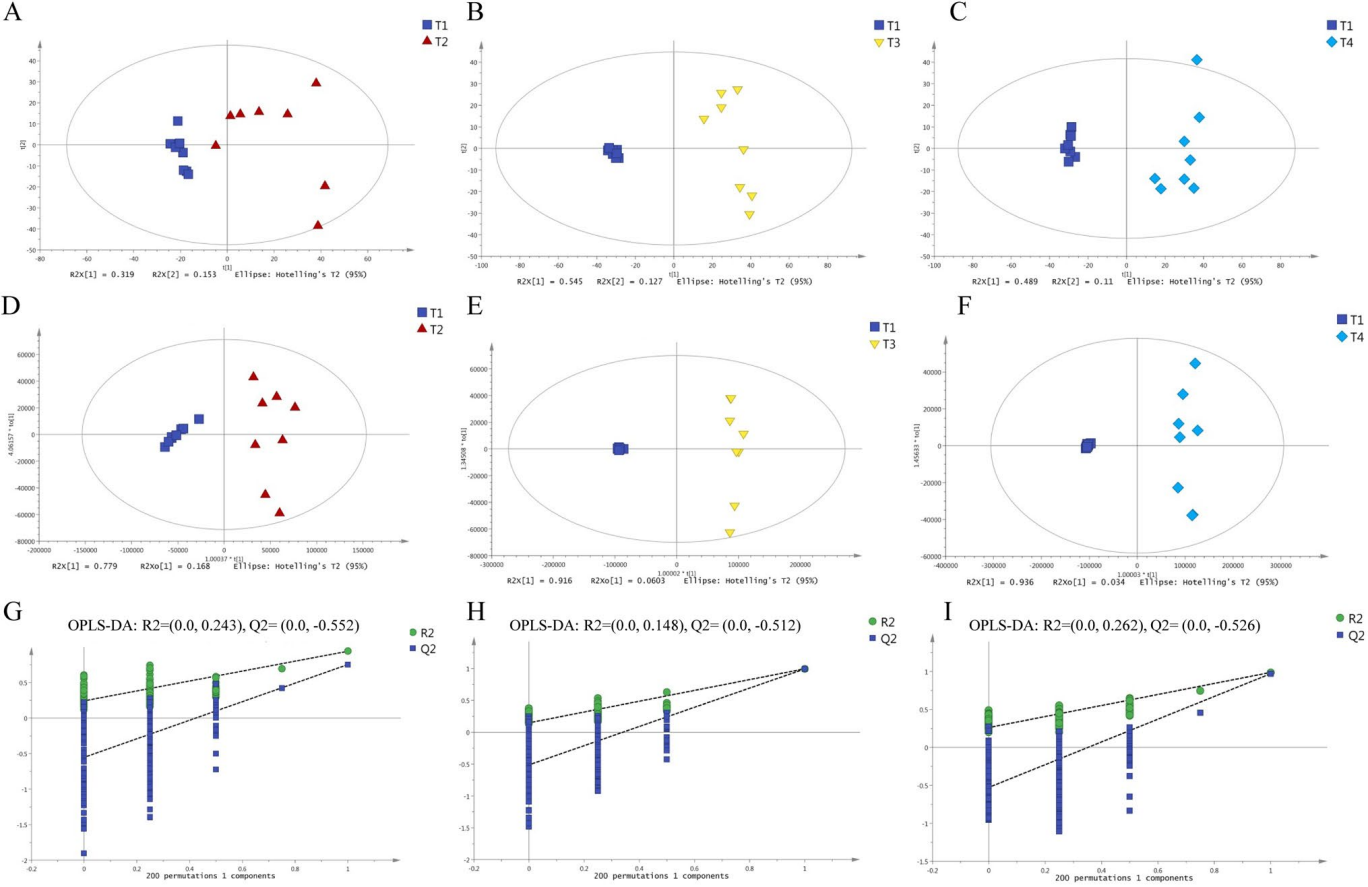
Analysis of lipid accumulation at different stages of kernel development in *Paeonia ostii.* (**A**–**C**) PCA plots, showing (**A**) T1 as compared to T2, (**B**) T1 as compared to T3, and (**C**) T1 as compared to T4. (**D**–**F**) OPLS-DA plots, showing (**D**) T1 as compared to T2, (**E**) T1 as compared to T3, and (**F**) T1 as compared to T4. (**G**–**I**) Permutation tests of the OPLS-DA plots (200 random permutations), showing (**G**) T1 as compared to T2, (**H**) T1 as compared to T3, and (**I**) T1 as compared to T4. R2 (downregulate squares) indicates the goodness of fit of each OPLS-DA model, while Q2 (green circles) reflects the predictive value of each model, based on the percentage of the correctly classified samples during cross-validation. Regression lines for R2 and Q2 are shown in each plot.

Progenesis QI qualitative analysis identified 1963 lipids: 1455 in positive ion mode and 508 in negative ion mode (Supplementary Table S2). Lipids significantly differentially abundant between developmental stages were analyzed further. PCA (Fig. 1A–F) and OPLS-DA (Fig. 1G–I) analyses of the Progenesis data matrix showed a clear distinction between samples at 35 DAF, when lipid accumulation was minimal (sample set T1), and samples taken at 49 DAF and later, when lipid accumulation was increasing significantly (Fig. 1A–F). Although samples at 49 DAF remained distinct from samples at 77 DAF and 119 DAF (Supplementary Fig. S2A,B,D,E), samples taken at 77 DAF were more difficult to distinguish from those taken at 119 DAF (Supplementary Fig. S2C,F). This may be because changes in lipid composition are more gradual towards the end of seed development. The permutation tests of the OPLS-DA models showed that the Q2 values of all samples were less than zero, indicating that model fit was good.

Lipid composition analysis identified 844 phospholipid species, accounting for 43% of the total lipid species (Supplementary Fig. S3). Within the phospholipids, we identified 216 phosphatidic acids (PAs), 189 phosphatidyl glycerols (PGs), 128 phosphatidyl serines (PSs), 123 PCs, 102 phosphatidyl inositols (PIs), 79 PEs, and 7 unclassified phospholipids. We also identified 479 neutral glycerides, accounting for 24% of the total lipid species. TAGs were the most common neutral glyceride, with 355 species (74% of the identified neutral glyceride species), followed by DAGs (117 species; 24.5%). FAs and their conjugates accounted for 47% of the 514 fatty acyl groups, with the remaining fatty acyl groups comprising alkanes, fatty aldehydes, fatty alcohols, fatty amides, and fatty acyl glycosides. We identified 89 sphingolipid species (4.5% of the total number of lipid species), 14 glycerol glycolipids, and 8 sterols. There were also 15 unclassified lipid components.

Lipid species were screened a second time, and those with fragmentation scores > 60 and scores > 45 were selected. Using this scoring system, we identified 56 TAGs (Table 1) and 64 PLs (Supplementary Table S2). The acyl chains of the 56 identified TAGs ranged from C13:0 to C22:0, and included UFAs (16:1, 18:1, 18:2, 18:3, 20:1, 20:2, 20:3, 20:4, 20:5, 22:1) and odd chains (13:0, 15:0, 17:0, 17:1; Table 1). TAGs were the most common lipid components in the *P. ostii* kernel, but PLs were the most common group overall. PLs are not only the main component of cellular membranes, but also provide a variety of fatty acyl chains for TAG synthesis^13^. The 64 phospholipid species identified using our scoring system included six phospholipid classes: PAs (31 molecular species), PCs (11 molecular species), PEs (10 molecular species), PGs (5 molecular species), PIs (4 molecular species), and PSs (3 molecular species; Supplementary Table S2). The predominant lipid component of FAs was PL; TAG was less abundant. The greatest PL losses were observed in PA, PC, and PE in the *P. ostii* seed. The lipid-species distribution pattern observed in tree peony differed from that observed in peanut (*Arachis hypogaea* L.)^26^: the greatest PL losses were observed in PE, PC, and PI in peanut seeds.

**Table 1:** Molecular species of triacylglycerols (TAGs) identified in the *Paeonia ostii* seed kernel.

Lipid name (C:N)	m/z	t_R_ (min)	Adducts	Acyl chain composition
TAG (47:0)	768.7063	13.8955	M + NH_4_	13:0–15:0–16:0
TAG (49:0)	796.7382	14.2677	M + NH_4_	14:0–16:0–16:0
TAG (50:0)	831.6834	14.4577	M + Na	14:0–16:0–17:0
TAG (51:0)	824.7694	14.6701	M + NH_4_	14:0–16:0–18:0
TAG (51:1)	822.7522	14.2487	M + NH_4_	14:0–16:0–18:1
TAG (51:5)	814.6909	12.9017	M + NH_4_	12:0–18:2–18:3
TAG (51:6)	833.6054	12.6178	M + NH_4_	12:0–18:3–18:3
TAG (52:0)	859.7143	14.8440	M + NH_4_	15:0–17:0–17:0
TAG (52:1)	836.769	14.4577	M + NH_4_	15:0–17:0–17:1
TAG (52:5)	828.7065	13.1074	M + NH_4_	13:0–18:2–18:3
TAG (53:0)	852.8001	15.0562	M + NH_4_	15:0–17:0–18:0
TAG (53:1)	850.7842	14.6507	M + NH_4_	16:0–16:0–18:1
TAG (53:2)	848.7688	14.3062	M + NH_4_	16:0–16:1–18:1
TAG (53:3)	851.71	12.4640	M + Na	16:0–16:1–18:2
TAG (53:4)	865.6667	13.5680	M + Na	14:0–18:1–18:3
TAG (53:5)	863.6513	13.2498	M + K	14:0–18:2–18:3
TAG (53:5)	842.7213	13.2692	M + NH_4_	14:0–18:2–18:3
TAG (53:6)	840.7052	12.9788	M + NH_4_	14:0–18:3–18:3
TAG (54:1)	885.7299	14.8441	M + NH_4_	16:0–17:0–18:1
TAG (54:2)	862.7851	14.4577	M + NH_4_	16:0–17:1–18:1
TAG (54:3)	860.7686	14.0948	M + NH_4_	15:0–18:1–18:2
TAG (54:4)	858.7531	13.7992	M + NH_4_	15:0–18:1–18:3
TAG (54:5)	856.7375	13.4725	M + NH_4_	15:0–18:2–18:3
TAG (54:6)	854.7216	13.1920	M + NH_4_	15:1–18:2–18:3
TAG (55:1)	878.8161	15.017	M + NH_4_	16:0–18:0–18:1
TAG (55:3)	874.7849	14.2875	M + NH_4_	16:0–18:1–18:2
TAG (55:4)	872.7692	13.9719	M + NH_4_	16:0–18:1–18:3
TAG (55:5)	875.7102	12.1935	M + Na	16:1–18:1–18:3
TAG (55:5)	870.7533	13.6638	M + NH_4_	16:0–18:2–18:3
TAG (55:6)	1724.404	13.405	2 M + Na	16:0–18:3–18:3
TAG (55:6)	873.6945	11.8679	M + Na	16:1–18:2–18:3
TAG (56:5)	884.7693	13.8373	M + NH_4_	17:0–18:2–18:3
TAG (56:6)	882.7536	13.5291	M + NH_4_	17:1–18:2–18:3
TAG (57:3)	902.8166	14.6312	M + NH_4_	18:0–18:1–18:2
TAG (57:4)	905.7552	14.2677	2 M + NH4	18:1–18:1–18:2
TAG (57:4)	900.8007	14.2487	M + K	18:0–18:2–18:2
TAG (57:5)	898.785	13.9145	M + K	18:1–18:1–18:3
TAG (57:6)	917.6981	13.5875	M + K	18:1–18:2–18:3
TAG (57:8)	1772.406	13.0163	2 M + Na	18:2–18:3–18:3
TAG (57:9)	1768.374	12.7329	2 M + Na	18:3–18:3–18:3
TAG (58:2)	939.7785	15.1909	M + NH_4_	18:0–18:1–19:1
TAG (58:7)	908.7688	13.5101	M + NH_4_	17:0–18:3–20:4
TAG (58:8)	906.7535	13.2116	M + NH_4_	17:0–18:3–20:5
TAG (59:2)	953.7926	15.3830	M + NH_4_	18:1–18:1–20:0
TAG (59:3)	930.8482	15.017	M + NH_4_	18:1–18:2–20:0
TAG (59:4)	928.8324	14.7082	M + NH_4_	18:1–18:2–20:1
TAG (59:5)	926.8165	14.3802	M + NH_4_	18:2–18:3–20:0
TAG (59:5)	947.7449	14.3994	M + Na	18:1–18:3–20:1
TAG (59:6)	924.8004	14.0948	M + NH_4_	18:2–18:3–20:1
TAG (59:7)	922.7847	13.7066	M + NH_4_	18:3–18:3–20:1
TAG (59:7)	943.7134	13.7153	M + Na	16:0–20:2–20:5
TAG (61:3)	979.8085	15.4022	M + NH_4_	18:1–18:2–22:0
TAG (61:4)	956.863	15.0756	M + NH_4_	18:1–18:3–22:0
TAG (61:5)	959.8028	14.7667	M + Na	18:2–18:3–22:0
TAG (61:6)	952.8312	14.4577	M + NH_4_	18:3–18:3–22:0
TAG (61:7)	950.8148	14.0562	M + NH_4_	18:3–18:3–22:1

Lipid compounds were selected using a scoring system. First, lipids were scored, based on accurate mass numbers, secondary fragments, and isotope distributions. Each of these criteria were assigned a maximum potential score of 20 points to yield a maximum potential total score of 60. Second, because the mass spectra of the secondary fragments are relatively important, we also calculated a fragmentation score (maximum 100 points). We then identified the TAG molecular species with fragmentation scores > 60 and scores > 45.

C:N represents the total number of carbons (C) and the number of double bonds (N). t_R_ indicates retention time. The fatty acyl chains of the listed TAG molecular species were assigned based on the major FA-fragment ions observed in the MS/MS spectra of each TAG. Details of the listed TAG molecular species are given in Supplementary Table S1.

Several FAs not commonly detected in tree peony seeds were identified. LC–MS/MS data analysis identified odd FAs (saturated and unsaturated), including 13:0, 15:0, 15:1, 17:0, 17:1 and 21:0, on TAG and polar lipids (Table 1 and Supplementary Table S2). Unusual odd FAs are known to be present in plants, bacteria, filamentous fungi, yeasts, algae, and protozoa^27^. Alves et al.^28^ also detected odd FAs, including 13:0, 15:0, 15:1, 17:0, 17:1, 19:0, and 21:0, on TAG and polar lipids of olive (*Olea europaea* L.) by LC–MS/MS.

**Figure 2:**
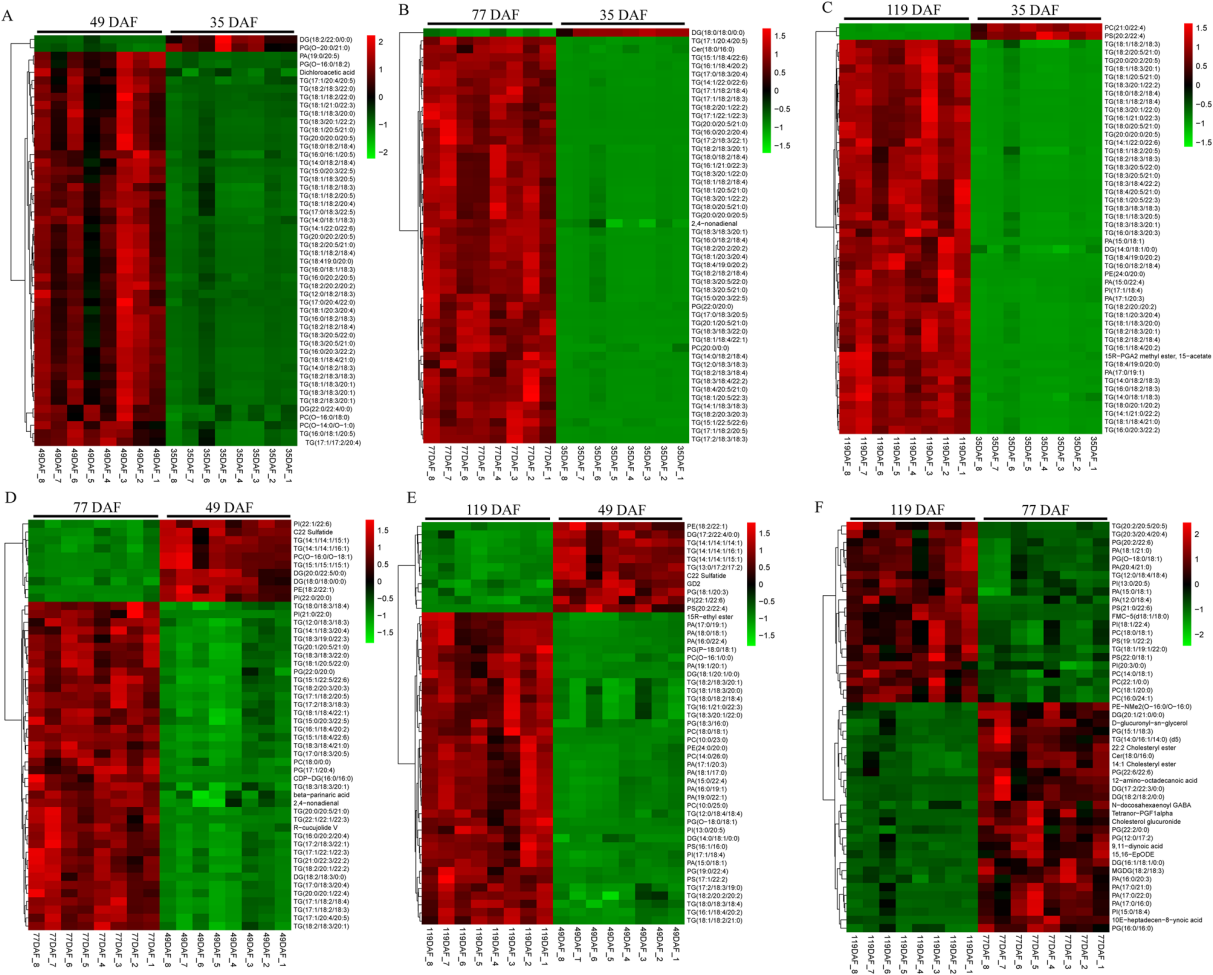
Hierarchal-clustering heatmaps showing differences in lipid abundance (based on VIP value) between developmental stages in the *Paeonia ostii* kernel.

We selected the lipid components with the 50 highest VIP values, corresponding to those that were the most differentially abundant between pairs of sample sets (T1–4). We then used hierarchal-clustering heatmaps, which clearly and intuitively show differences in lipid abundance among groups, to visualize differences in lipid abundance among sample sets. The heat maps showed that the abundance patterns of the lipid components with VIP values > 50 changed significantly between adjacent kernel-developmental stages (Fig. 2). The differences were most noticeable between the period before lipid accumulation (35 DAF; sample set T1) and the subsequent periods of lipid accumulation (49–119 DAF; sample sets T2–4; Fig. 2A–C). As the end of lipid accumulation approached, the differences in lipid abundances between adjacent stages became less pronounced (Fig. 2F). These results indicated that the lipid metabolism was most active during the rapid seed-oil accumulation period (49–77 DAF). Consistent with this, our Pearson correlation analysis identified more significant correlations between pairs of significantly differentially abundant lipids in different subclasses between adjacent developmental stages at the beginning of lipid accumulation (Supplementary Fig. S4A–D) as compared to the end of lipid accumulation (Supplementary Fig. S4E–F). Correlation analyses can help to quantify the relationships between pairs of significantly differentially abundant lipid molecules, in order to clarify the processes of biological state change.

### FA composition and distributions of major lipids in the *P. ostii* kernel

To better understand the variations in the FA composition of lipids during seed development, we characterized differences in the molecular species comprising TAG, DAG, PA, PC, and PE among the four developmental stages.

**Figure 3:**
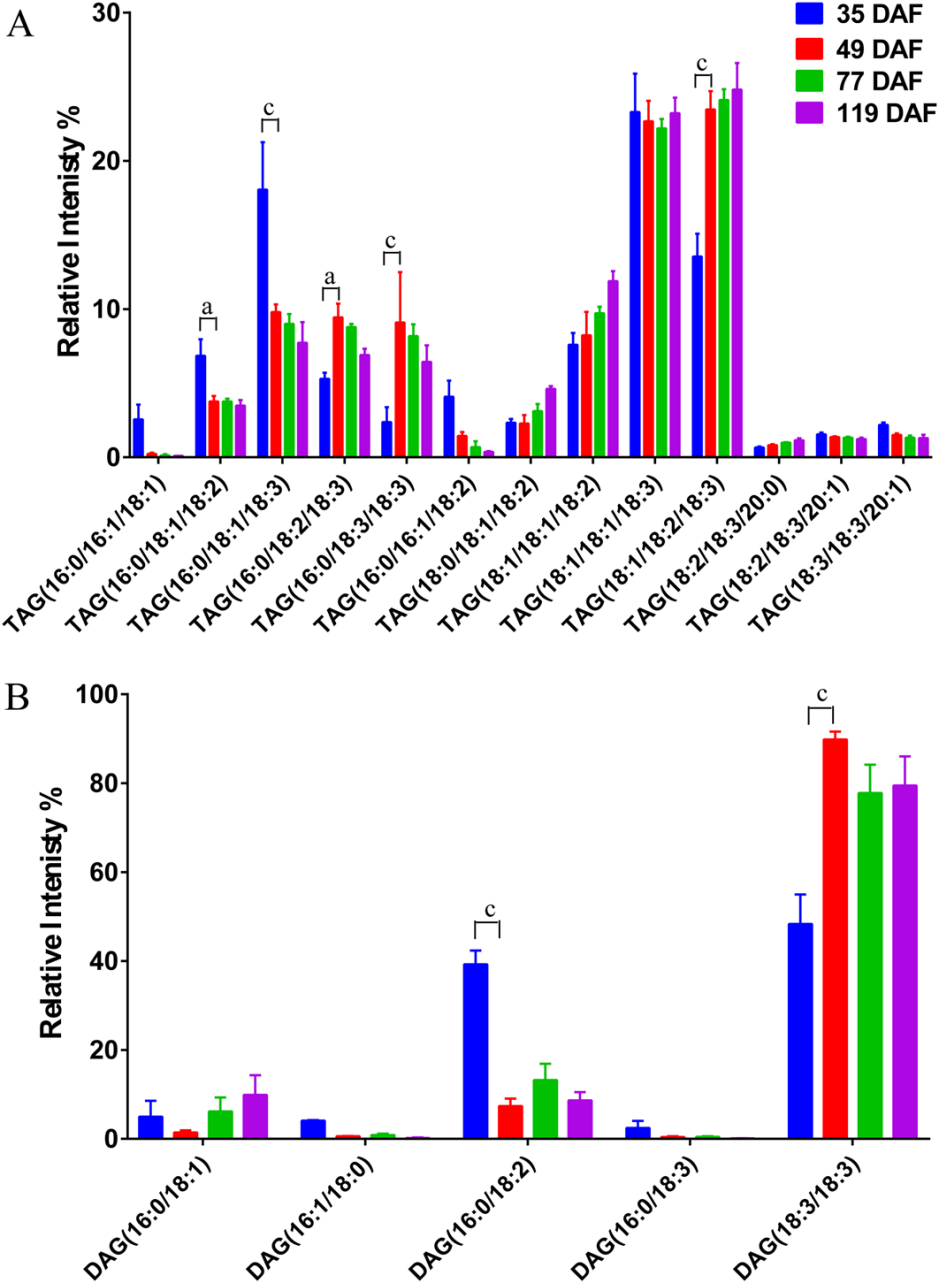
Changes in the relative abundances of the major molecular species comprising (**A**) triacylglycerol (TAG) and (**B**) diacylglycerol (DAG) during the development of the *Paeonia ostii* kernel. DAF, days after fertilization. Means ± SD (n = 8) are shown. Lowercase letters correspond to significant differences between adjacent developmental stages: a, *p* < 0.05; b, *p* < 0.01; c, *p* < 0.001. Only molecular species representing > 0.5% of total abundance are shown.

Our FA composition analysis showed that UFAs were the main molecular species comprising TAGs. This was consistent with a previous report, which stated that UFAs accounted for more than 90% of all FAs in seeds^3^. The most abundant acyl chains in TAG at all time points were 18:1/18:2/18:3, 18:1/18:1/18:3, 18:1/18:1/18:2, 16:0/18:3/18:3, 16:0/18:1/18:3, 16:0/18:2/18:3, and 16:0/18:1/18:3 (Fig. 3A). Between 35 and 49 DAF, the relative contents of several UFAs changed significantly: the abundances of 18:1/18:2/18:3, 18:1/18:1/18:2, 16:0/18:3/18:3, and 16:0/18:2/18:3 increased significantly, while those of 16:0/18:1/18:2 and 16:0/18:1/18:3 decreased significantly (Fig. 3A). DAG was composed of fewer different molecules species, most commonly 18:3/18:3 and 16:0/18:2 (Fig. 3B). The relative abundance of 18:3/18:3 increased significantly between 35 and 49 DAF, while that 16:0/18:2 decreased significantly (Fig. 3B). During the period 35–49 DAF, the most abundant species in TAG were those containing single saturated FAs or more highly unsaturated species (18:1/18:1/18:3, 18:1/18:2/18:3), consistent with the fatty acid composition in tree peony seed^3,4,16,17^. However, the next most abundant TAG species, which were those containing palmitate (16:0/18:1/18:3, 16:0/18:1/18:2, and 16:0/16:1/18:1), decreased in abundance during the period 35–49 DAF. Indeed, there was a general shift toward more unsaturated TAG molecular species during the oil accumulation period (Fig. 3). A shift toward more unsaturated TAG molecular species during the oil accumulation period of seed development was also observed in other oil seed crops, such as soybean (*Glycine max* L.)^29^ and rape (*Brassica napus* L.)^25^.

Our analysis showed that the composition of PAs, PCs, and PEs in the *P. ostii* kernel also changed greatly over the four stages of kernel development (Supplementary Fig. S5). At 35 DAF, when oil accumulation was minimal, the most common FAs in PA included 16:0/18:2, 18:1/18:3, and 18:2/18:1 (Supplementary Fig. S5A). However, during the period of rapid oil accumulation (between 35 and 77 DAF), the relative contents of these FAs decreased significantly, while the relative contents of 17:0/18:2 increased significantly (Supplementary Fig. S5A). PC and PE are acyl carriers for FA acyl editing in the ER^13^. Based on the molecular species identified in PC and PE, PC should be the main acyl carrier for FA editing during the kernel development in *P. ostii*. The most common FAs in PC were 16:0/18:1, 16:0/20:4, 18:1/18:1, and 18:1/18:2 (Supplementary Fig. S5B). The contents of 16:0/18:1 and 18:1/18:1 increased significantly during development, while the contents of 16:0/20:4 and 18:1–18:2 decreased significantly (Supplementary Fig. S5B). Levels of 18:3 in PC were very low across all four developmental stages (Supplementary Fig. S5B). Transcriptome analysis of tree peony seed development indicated that FAD3 was the primary enzyme catalyzing the desaturation of 18:2 to 18:3^17,19,20^. This implied that 18:3 should be abundant in PC. However, our results showed that levels of 18:3 were very low in PC. PC can contribute a fatty acid directly into TAG via PDAT^15,30^. Thus, we speculated that PC is directly used as donor to form TAG (18:3) via PDAT in *P. ostii*. The results of the tree-peony lipidomic analysis were consistent with the results of the tree-peony transcriptome analysis. A previous study, which used lipidomics to reveal the details of lipid accumulation in the developing seeds of oilseed rape, suggested that DGAT may have important effects on the molecular composition of TAG^25^.

### KEGG pathway and metabolic network analysis

**Figure 4:**
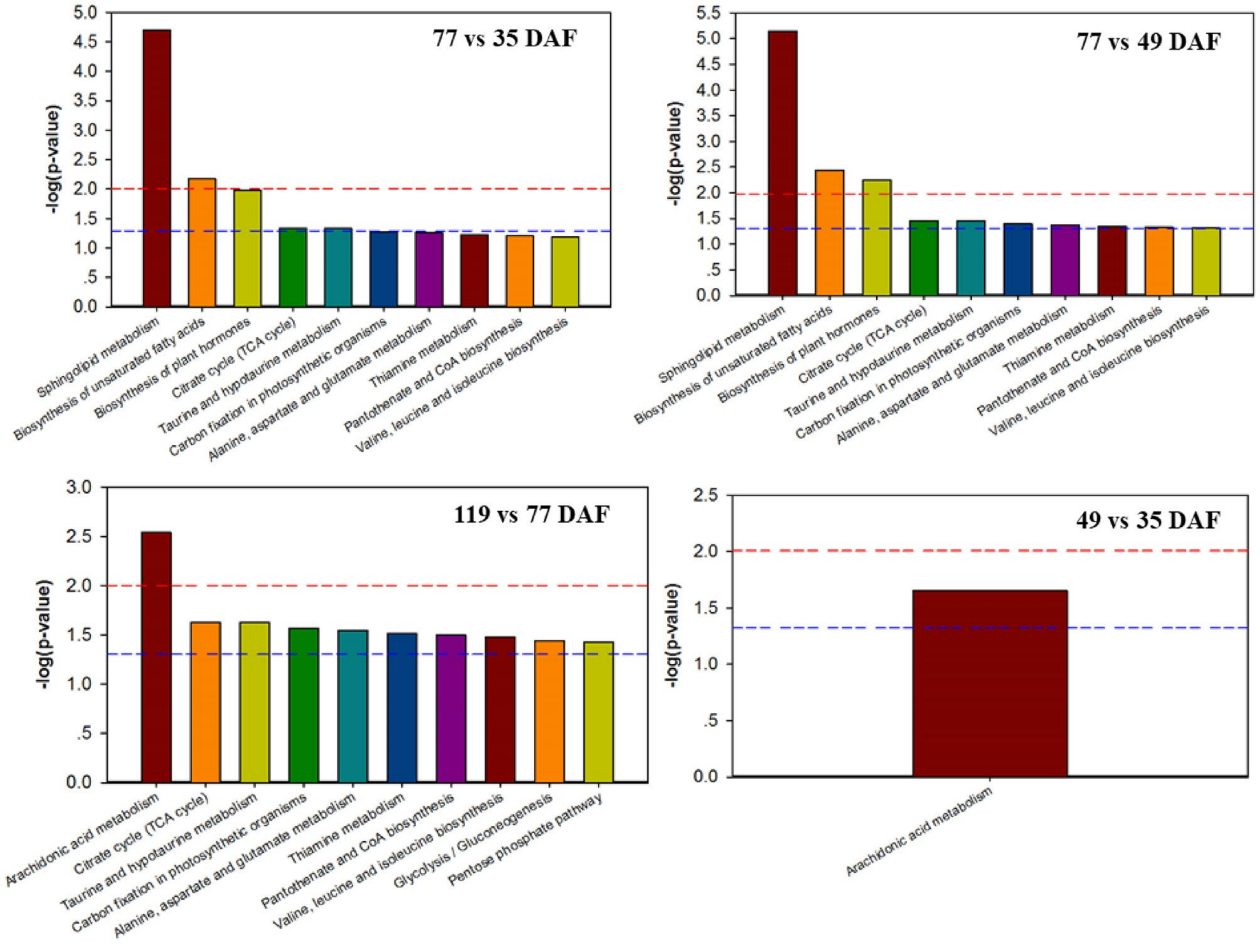
The 10 KEGG pathways most significantly overenriched in the lipid components differentially abundant during kernel development in *Paeonia ostii*.

Several lipid components were identified as significantly differentially abundant between adjacent developmental stages (*P* < 0.05; Table 2). We identified several KEGG pathways that were significantly enriched in these lipid components (*P* < 0.05; Fig. 4). For example, three metabolic pathways were associated with the lipid components differentially abundant between 77 and 49 DAF: sphingolipid metabolism, biosynthesis of UFAs, and biosynthesis of plant hormones (Fig. 4B). Only one metabolic pathway (arachidonic acid metabolism) was significantly enriched in the metabolites differentially abundant at 49 DAF as compared to 35 DAF. Other metabolic pathways overrepresented in the differentially abundant metabolites included the citrate cycle (TCA cycle); the taurine and hypotaurine metabolism; carbon fixation in photosynthetic organisms; the alanine, aspartate, and glutamate metabolism; the thiamine metabolism; pantothenate and CoA biosynthesis; valine, leucine, and isoleucine biosynthesis; glycolysis/gluconeogenesis; and the pentose phosphate pathway (Fig. 4).

**Table 2:** Lipids significantly differentially abundant in the *Paeonia ostii* kernel throughout seed development (35–119 days after fertilization, DAF).

Metabolites	log2 (FC)	KEGG	Expression
**49 DAF vs 35 DAF (T2 vs T1)**			
Alpha,alpha'-Trehalose 6-mycolate	4.08496	C04218	Up
(2S,3S)-2-hydroxytridecane-1,2,3-tricarboxylic acid	− 1.4214	C04655	Down
13R-HODE	1.73666	C14762	Up
PGD1	2.66062	C06438	Up
LTB4-d4	1.28712	C02165	Up
**77 DAF vs 35 DAF (T3 vs T1)**			
(9S,10S)-10-hydroxy-9-(phosphonooxy)octadecanoic acid	13.6398	C15989	Up
Stearic acid	28.5099	C01530	Up
Alpha-linolenic acid	28.0321	C06427	Up
13,16,19-docosatrienoic acid	− 1.1	C16534	Down
Pyruvic acid	− 4.2735	C00022	Down
alpha,alpha'-Trehalose 6-mycolate	6.21813	C04218	Up
Azelaic acid	− 1.4218	C08261	Down
(2S,3S)-2-hydroxytridecane-1,2,3-tricarboxylic acid	− 5.2261	C04655	Down
13R-HODE	4.79309	C14762	Up
7S,8S-DiHOTrE	7.02643	C07357	Up
PGD1	5.35498	C06438	Up
LTB4-d4	1.07959	C02165	Up
Palmitoylcarnitine	1.35008	C02990	Up
Sphingosine-1-phosphate	− 27.186	C06124	Down
Sphinganine-phosphate	1.12478	C01120	Up
C22 Sulfatide	− 3.1302	C06125	Down
**119 DAF vs 35 DAF (T4 vs T1)**			
(9S,10S)-10-hydroxy-9-(phosphonooxy)octadecanoic acid	14.3746	C15989	Up
Tridecylic acid	− 1.3686	C13795	Down
Stearic acid	29.7946	C01530	Up
Alpha-linolenic acid	27.8896	C06427	Up
Pyruvic acid	− 9.1204	C00022	Down
Alpha,alpha'-Trehalose 6-mycolate	7.0312	C04218	Up
(2S,3S)-2-hydroxytridecane-1,2,3-tricarboxylic acid	− 7.738	C04655	Down
13R-HODE	1.96873	C14762	Up
20-HETE-d6	− 2.0994	C14748	Down
Palmitoylcarnitine	1.72443	C02990	Up
sn-3-O-(geranylgeranyl)glycerol 1-phosphate	− 3.3033	C04590	Down
Sphingosine-1-phosphate	− 27.186	C06124	Down
Sphinganine-phosphate	1.21741	C01120	Up
C22 Sulfatide	− 3.3901	C06125	Down

Compared with other periods, stearic acid (SA) and ALA were significantly up-regulated at 77–119 DAF as compared to 35 DAF, while palmitoylcarnitine and sphinganine-phosphate were slightly less abundant. Conversely, pyruvic acid, sphingosine-1-phosphate, and sulfonate were down-regulated at 77–119 DAF as compared to 35 DAF (Table 2); in particular, sphingosine-1-phosphate was significantly down-regulated during the oil accumulation stages (77–119 DAF; Table 3). During oil accumulation and kernel development, levels of both ALA and its substrate, SA, increased significantly. It was thus unsurprising that the SA pathway was overrepresented in many of the metabolites differentially abundant during the rapid oil-accumulation stages. SA is an octadecanoic fatty acid, and octadecanoic fatty acids are the initial substrates for oil synthesis and accumulation^12^. Consistent with this, octadecanoic acid was the most accumulated FA in the *P. ostii* kernel. In this study, SA content only indicated the content of the free fatty acid. Our results showed that the content of free SA changed significantly in the developing kernel of *P. ostii*. In other studies^16,17^, SA content corresponds to SA content after the methyl esterification of all lipids. Therefore, lipidomics is a better method for qualitative and quantitative lipid analysis.

**Table 3:** Lipids significantly differentially abundant in the *Paeonia ostii* kernel throughout seed development (49–119 days after fertilization, DAF).

Metabolites	log2 (FC)	KEGG	Expression
**77 DAF vs 49 DAF (T3 vs T2)**			
(9S,10S)-10-hydroxy-9-(phosphonooxy)octadecanoic acid	30.4281	C15989	Up
Stearic acid	4.02473	C01530	Up
Alpha-linolenic acid	28.0321	C06427	Up
13,16,19-docosatrienoic acid	− 1.3686	C16534	Down
Pyruvic acid	− 3.6694	C00022	Down
Alpha,alpha'-Trehalose 6-mycolate	2.13317	C04218	Up
Azelaic acid	− 1.3931	C08261	Down
(2S,3S)-2-hydroxytridecane-1,2,3-tricarboxylic acid	− 3.8047	C04655	Down
13R-HODE	3.05643	C14762	Up
7S,8S-DiHOTrE	2.88398	C07357	Up
PGD1	2.69436	C06438	Up
Sphingosine-1-phosphate	− 28.47	C06124	Down
Sphinganine-phosphate	1.10684	C01120	Up
C22 Sulfatide	− 2.723	C06125	Down
**119 DAF vs 49 DAF (T4 vs T2)**			
(9S,10S)-10-hydroxy-9-(phosphonooxy)octadecanoic acid	31.1629	C15989	Up
Tridecylic acid	− 1.3921	C13795	Down
Stearic acid	5.30942	C01530	Up
Alpha-linolenic acid	27.8896	C06427	Up
Pyruvic acid	− 8.5163	C00022	Down
Alpha,alpha'-Trehalose 6-mycolate	2.94624	C04218	Up
(2S,3S)-2-hydroxytridecane-1,2,3-tricarboxylic acid	− 6.3166	C04655	Down
6-keto PGE1	− 2.9748	C05962	Down
Palmitoylcarnitine	1.23672	C02990	Up
sn-3-O-(geranylgeranyl)glycerol 1-phosphate	− 4.213	C04590	Down
Sphingosine-1-phosphate	− 28.47	C06124	Down
Sphinganine-phosphate	1.19947	C01120	Up
C22 Sulfatide	− 2.983	C06125	Down
**119 DAF vs 77 DAF (T4 vs T3)**			
Stearic acid	1.28469	C01530	Up
Pyruvic acid	− 4.8469	C00022	Down
2-amino-4-cyano-butanoic acid	1.10712	C05717	Up
13R-HODE	− 2.8244	C14762	Down
7S,8S-DiHOTrE	− 2.7318	C07357	Down
6-keto PGE1	− 2.7313	C05962	Down
PGD1	− 2.5711	C06438	Down
20-HETE-d6	− 2.682	C14748	Down
sn-3-O-(geranylgeranyl)glycerol 1-phosphate	− 3.2898	C04590	Down

Pyruvic acid, the final product of glycolysis, realizes the transformation of carbohydrates and lipids via acetyl-CoA and the citrate cycle^31,32^. Pyruvate is pivotal for the metabolic connections among these nutrients^33,34^. During rapid oil accumulation in *P. ostii* (77–119 DAF), pyruvic acid abundance decreased significantly, possibly because it was used in multiple metabolic pathways, including the citrate cycle, carbon fixation in photosynthetic organisms, and glycolysis/gluconeogenesis (Supplementary Figs S6–S8). The significant decrease in pyruvic acid abundance thus reflected the rapid increase the abundances of some other substances, including lipids and carbohydrates. During kernel development in *P. ostii*, we therefore speculate that rapid lipid accumulation leads to a decrease in pyruvate content, revealing the mutual carbon flux among metabolic pathways.

The sphingolipid metabolic pathway was significantly enriched in many of the metabolites differentially abundant during the rapid oil-accumulation stages (Fig. 4A–B). Among all significantly enriched pathways, the sphingolipid metabolic pathway was the most significant (Fig. 4). Sphingolipids and their metabolites are very important active molecules, which participate in a variety of physiological and biochemical reactions in cells, and strengthen the tolerance or adaptation of plants to various adversity environments, such as tolerance to drought, heavy metals, chilling injury and cold adaptation^35^. Sphingosine-1-phosphate content decreased significantly during oil accumulation (77–119 DAF) as compared to the period of rapid growth before oil accumulation (before 49 DAF); during the oil accumulation period, sphinganine-phosphate content increased slightly (Table 2). Sphingosine-1-phosphate stimulates cell proliferation and differentiation during plant growth and development^36^. Therefore, the decrease in sphingosine-1-phosphate between fast growth and oil accumulation suggested that cell proliferation and differentiation in the seed kernel ceased during oil accumulation.

## Conclusion

The lipids extracted from *P. ostii* seeds were analyzed using UPLC-Q-TOF–MS/MS to characterize FA abundances and distributions throughout kernel development. PLs and TAG were the most abundant lipids in the *P. ostii* kernel throughout development, followed by fatty acyls, sphingolipids, and glycolipids. TAG was comprised of the greatest variety of FA chains, primarily UFAs, including 18:1/18:2/18:3, 18:1/18:1/18:3, 18:1/18:1/18:2, 16:0/18:3/18:3, 16:0/18:2/18:3, 16:0/18:1/18:3, and odd-carbon FAs (e.g., 15:0/17:0/17:0, 15:0/17:0/18:0, and 17:1/18:2/18:3). The abundances of 18:1, 18:2, and 18:3 in TAG acyl chains increased significantly during the rapid oil-accumulation stage in the kernel. Fewer different FAs were identified in PA and PC than in TAG, although more odd-carbon FAs were identified in PA. During seed development, monounsaturated FAs in PC and PE increased, while PUFAs decreased. The types and contents of lipid metabolites differed significantly among the different stages of kernel development; lipid distributions seemed to be specific to the developmental stage. Lipid-related metabolites, such as sphingosine-1-phosphate, pyruvic acid, stearic acid, and ALA, were significantly differentially abundant during the critical period of oil accumulation (49–119 DAF), and the corresponding metabolic pathways were significantly enriched. Notably, the sphingolipid metabolic pathways were significantly enriched during rapid seed-oil accumulation, but not significantly enriched in other periods. Our results provide a basis for future studies of the lipid metabolism and its regulation in tree peonies.

## Materials and methods

### Seed materials

The seeds were collected from mature *P. ostii* trees in Shanghai Chenshan Botanical Garden, Shanghai, China (31°4′52″N, 121°10′14″E). These *P. ostii* were introduced to the garden more than 10 years ago, and have been grown under the same environmental and cultivation conditions since that time. Now, there are thousands of living plants of *P. ostii* growing in Shanghai Chenshan Botanical Garden. We observed the development of the seeds produced by *P. ostii* trees. The budded flowers of four selected plants (CS03, CS09, CS15, and CS16) were hand-pollinated with pollen collected from a fifth *P. ostii* tree (CS10). The pollination date was designated 0 days after fertilization (DAF). Between 0 and 119 DAF, seeds were collected every seven days from each plant (a total of 17 samples/tree). After seed collection, the kernels were manually extracted, frozen in liquid nitrogen, and stored at − 80 °C. In addition, the voucher specimens of *P. ostii* was identified by Yonghong Hu and deposited in Shanghai Chenshan Herbarium numbered CSH0184197.

### Measurement of total lipids

To explore how total lipids changed throughout kernel development, we measured the lipids contents in kernel samples taken from the four selected plants between 35 and 119 DAF (a total of 27 samples/tree). Total lipids were extracted from the dried biomass as previously described^3^. Based on preliminary results, four stages of kernel development that differed significantly with respect to lipid accumulation were selected for further analysis: 35 DAF (sample set T1), 49 DAF (sample set T2), 77 DAF (sample set T3), and 119 DAF (sample set T4). Each sample set included eight replicates (a total of 32 samples). Two samples from each of the four trees listed above were taken.

### Sample preparation

We transferred 50 mg of each sample from sample sets T1–4 (accurately weighted) to separate 1.5 mL Eppendorf tubes. Two small steel balls were added to each tube, along with 20 μL of 2-chloro-1-phenylalanine (0.3 mg/mL), 20 μL of heptadecanoic acid (0.01 mg/mL) dissolved in methanol (internal standard), and 500 μL of water. All samples were incubated at − 20 °C for 2 min, and then ground at 60 Hz for 2 min. Each homogenate was transferred to a 10 mL glass centrifuge tube, 1.5 mL of a mixture of dichloromethane and methanol (v:v = 2:1) was added, and the tube was vortexed for 1 min. Each tube was chilled at − 20 °C for 20 min and then centrifuged at 1500 g and 4 °C for 15 min. The bottom layer of the stratified liquid was transferred to a new LC–MS injection vial, and evaporated in a vacuum. Next, this process was repeated using the remaining stratified liquid in the glass centrifuge tube: 1.5 mL of a mixture of dichloromethane and methanol (v:v = 2:1) was added to the stratified liquid in each tube, and the tubes were vortexed for 1 min. The tubes were then chilled at − 20 °C for 20 min. Chilled samples were centrifuged at 1500 g and 4 °C for 15 min. The bottom layer of the stratified liquid in each was transferred to the LC–MS vial containing the first extraction and allowed to dry out. After volatilization, the lipid residue in each LC–MS vial was redissolved with 400 μL isopropanol acetonitrile (v:v = 1:1) by vortexing for 30 s, followed by ultrasonic extraction for 3 min. This solution was then transferred to a 1.5 mL tube and centrifuged for 10 min at 9000 g and 4 °C. After centrifugation, 100 μL of the supernatant was removed, diluted with 150 μL isopropanol-acetonitrile (v:v = 1:1), filtered through a 0.22 μm microfilter, and transferred to a LC–MS injection vial. All injection vials were stored at − 80 °C until LC–MS analysis.

Samples from each group were mixed in equal amounts to generate a quality control (QC) sample. One QC sample was analyzed every eight samples, and the results of these analyses were used to evaluate the stability of the system throughout the experiment.

### UPLC-Q-TOF–MS/MS analysis

A Nexera UPLC (Shimadzu, Kyoto, Japan) system, fitted with a Q-Exactive quadrupole-Orbitrap mass spectrometer equipped with a heated electrospray ionization (HESI) source (Thermo Fisher Scientific, Waltham, MA, USA), was used to analyze the metabolic profiles in both ESI-positive and ESI-negative ion modes. ACQUITY UPLC BEH C18 columns (1.7 μm, 2.1 × 100 mm) were used in both positive and negative modes. The binary gradient elution system consisted of a 6:4 solution of acetonitrile–water (solution A; containing 0.1% formic acid and 10 mmol/L ammonium formate, v/v) and a 1:9 solution of acetonitrile-isopropanol (solution B; containing 0.1% formic acid and 10 mmol/L ammonium formate, v/v). Separations were performed using the following gradient: 30% B for 3 min, 30–62% B from 3 to 5 min, 62–82% B from 5 to 15 min, 82–99% B from 15–16.5 min, hold at 99% B for 1.5 min, 99–30% B from 18 to 18.1 min, and hold at 30% B from 18.1 to 22 min. The flow rate was 0.35 mL/min, and the column temperature was 45 °C. All samples were kept at 4 °C throughout the analysis. The injection volume was 5 μL. The mass range of the instrument was set to m/z 120–1800. The ion spray voltage was set to 3500 V in the positive ion mode and to -3100 V in the negative ion mode. The heater temperature was set to 300 °C, and the capillary temperature was set to 320 °C. The sheath gas flow rate was 45 arb, the aux gas flow rate was 15 arb, and the sweep gas flow rate was 1 arb. The S-Lens RF Level was 50%.

### Raw data preprocessing and lipid metabolite identification

The acquired LC–MS raw data were analyzed using progenesis QI software (version 2.3; Waters Corporation, Milford, USA) with the following parameters: precursor tolerance set to 5 ppm; fragment tolerance set to 10 ppm, and retention time (RT) tolerance set to 0.02 min. Internal standard detection parameters were deselected for peak RT alignments, isotopic peaks were excluded from the analysis, the noise elimination level was set to 10.00, and the minimum intensity was set to 15% of base peak intensity. The matrix data were obtained using three dimensional datasets (m/z, peak RT, and peak intensity), and an RT-m/z pair was used as the identifier for each ion. The matrix was further reduced by removing any peaks missing values (ion intensity = 0) in more than 60% of the samples. An internal standard was used for data QC. Principal component analysis (PCA) and (orthogonal) partial least-squares-discriminant analysis (O)PLS-DA were performed to visualize metabolic alterations among experimental groups, after mean centering (Ctr) and Pareto variance (Par) scaling, respectively^37^. The Hotelling’s T2 region, shown as an ellipse in the score plot of each model, defines the 95% confidence interval of the modeled variation. Variable importance in the projection (VIP) ranks the overall contribution of each variable to the OPLS-DA model, and those variables with VIP > 1 are considered relevant for group discrimination [CITE]. We used the default eight-round cross-validation, with one-eighth of the samples excluded from the mathematical model in each round, to guard against overfitting.

Lipid identification was performed using progenesis QI Data Processing Software, based on public database Lipidmaps (v2.3) (http://www.lipidmaps.org/). Metabolites were considered to differ significantly among groups if the OPLS-DA-obtained variable influence on projection (VIP) values were > 1.0 and the *p*-values generated by two-tailed Student’s tests of the normalized peak areas were < 0.05.

### KEGG pathway analysis

Metabolites were identified as differentially abundant between developmental stages (sample sets T1–4) when absolute value of log2 (FC) > 1.5 and *P* < 0.05. The differentially abundant metabolites were mapped onto the Kyoto Encyclopedia of Genes and Genomes (KEGG) metabolic database (https://www.kegg.jp/kegg/pathway.html)^38^using the MBRole (http://csbg.cnb.csic.es/mbrole2/) for KEGG pathway analysis. Metabolites were considered significantly enriched in a KEGG pathway if *P* < 0.05. KEGG enrichment pathways and heat maps were constructed using OmicShare, which is a free online platform for data analysis (www.omicshare.com/tools). Visual analysis of the metabolite-associated metabolic pathways was performed using Mapman (version 3.6.0 RC1)^39,40^.

### Statistical analysis

All charts were drawn using Excel 2017, OmicShare, and GraphPad Prism 7.0. Image layout was performed using Adobe Illustrator CS6. Parameter data were analyzed using one-way ANOVAs in GraphPad Prism 7.0.

### Ethical standards

The experiments involving plants performed in this study complied with the laws of China.

## Supplementary Information

Supplementary Information 1.

Supplementary Information 2.
