# Supplementary material for: Lipidomic profiling of the developing kernel clarifies the lipid metabolism of Paeonia ostii
Source: Sci Rep. 2021 Jun 15;11:12605. doi: 10.1038/s41598-021-91984-9 (PMC8206221; doi:10.1038/s41598-021-91984-9)
Supplement: Supplementary file 1 — Supplementary Information 1. [file 41598_2021_91984_MOESM1_ESM.docx]

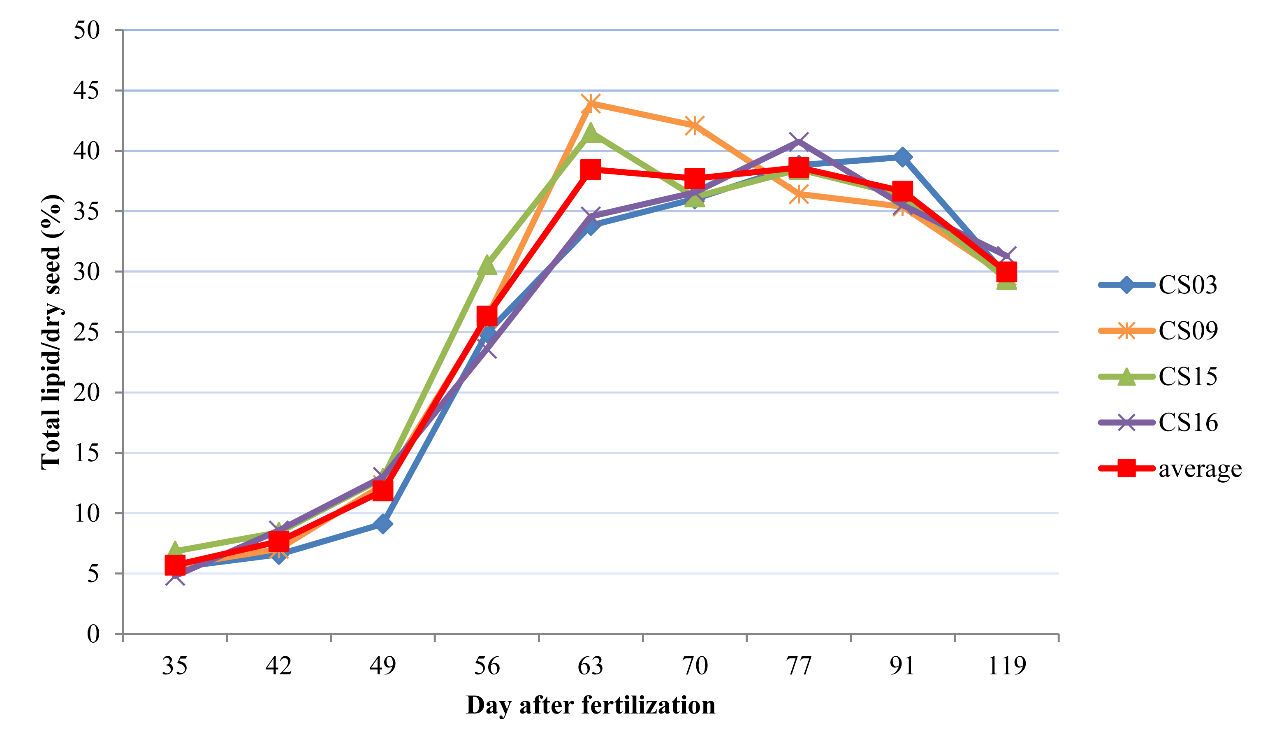


Supplementary Figure S1. Changes of total lipid content during kernel development in *Paeonia ostii*

CS03, CS09, CS15, CS16 represents 4 individual plants of *P*. *ostii* respectively.


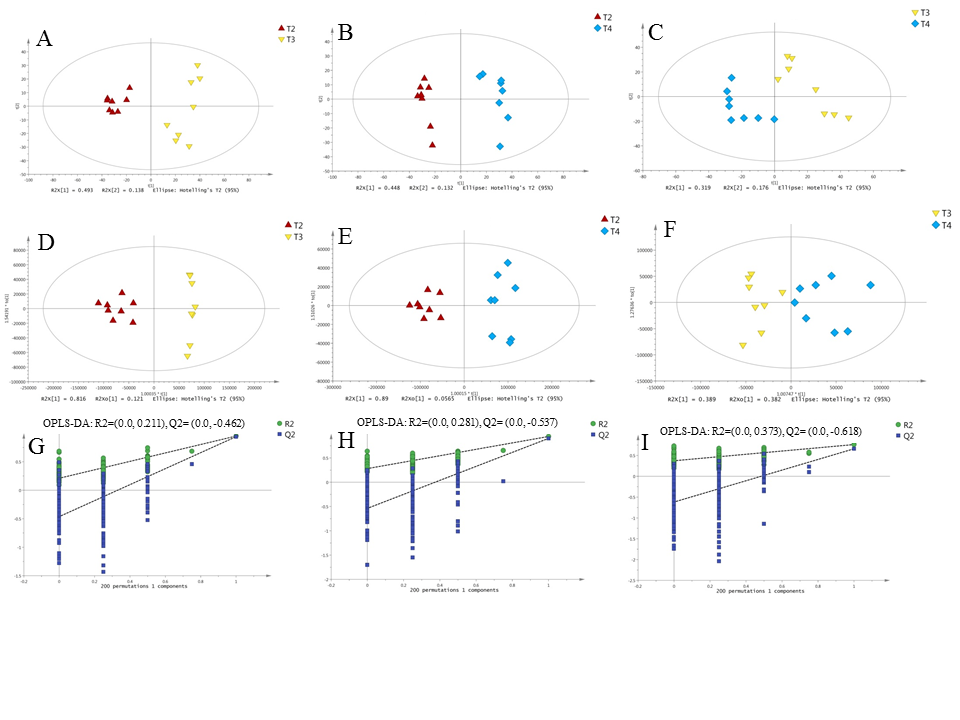


Supplementary Figure S2. Score plot of PCA and OPLS-DA models of kernel development in *Paeonia ostii*

(A, B, C) Score plot of PCA model, obtained from T3 vs T2, T4 vs T2, T4 vs T3; (D, E, F) Score plot of OPLS-DA model, obtained from T3 vs T2, T4 vs T2, T4 vs T3; (G, H, I) Score plot of OPLS-DA model, obtained from T3 vs T2, T4 vs T2, T4 vs T3.


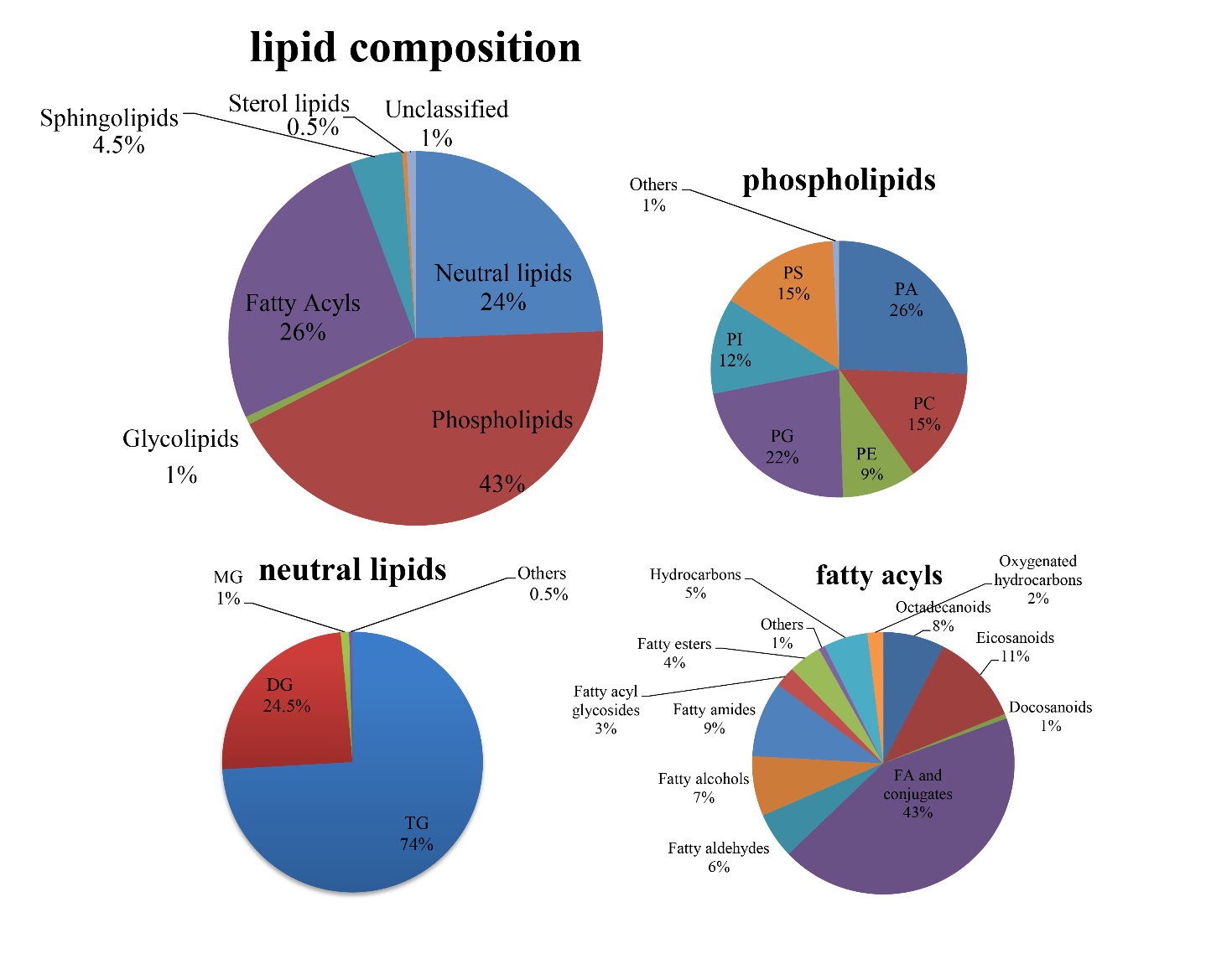


Supplementary Figure S3. The proportion of lipid components in the kernel of *Paeonia ostii*.


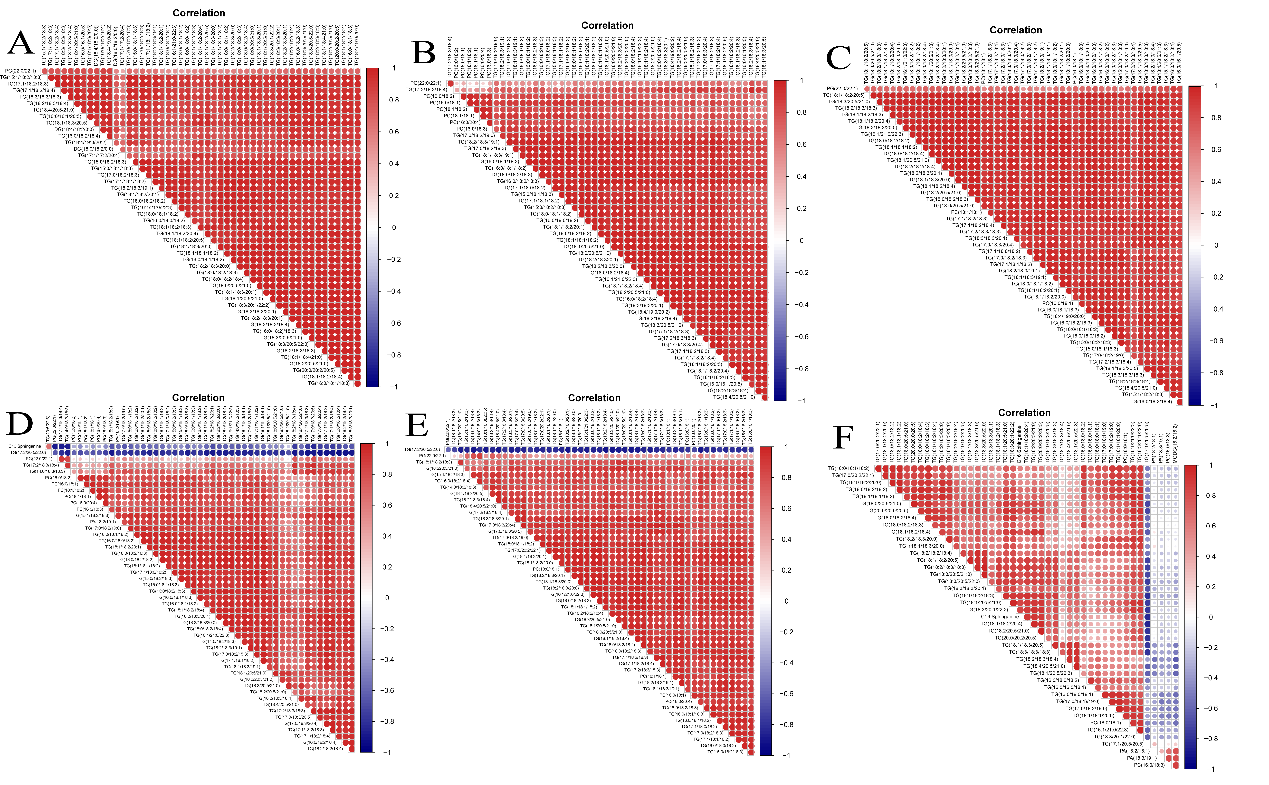


Supplementary Figure S4. Correlation analysis of Top-50 (VIP value) in four stages of *Paeonia ostii* kernel.

A, 49 DAF vs 35 DAF; B, 77 DAF vs 35 DAF; C, 119 DAF vs 35 DAF; D, 77 DAF vs 49 DAF; E, 119 DAF vs 49 DAF; F, 119 DAF vs 77 DAF.


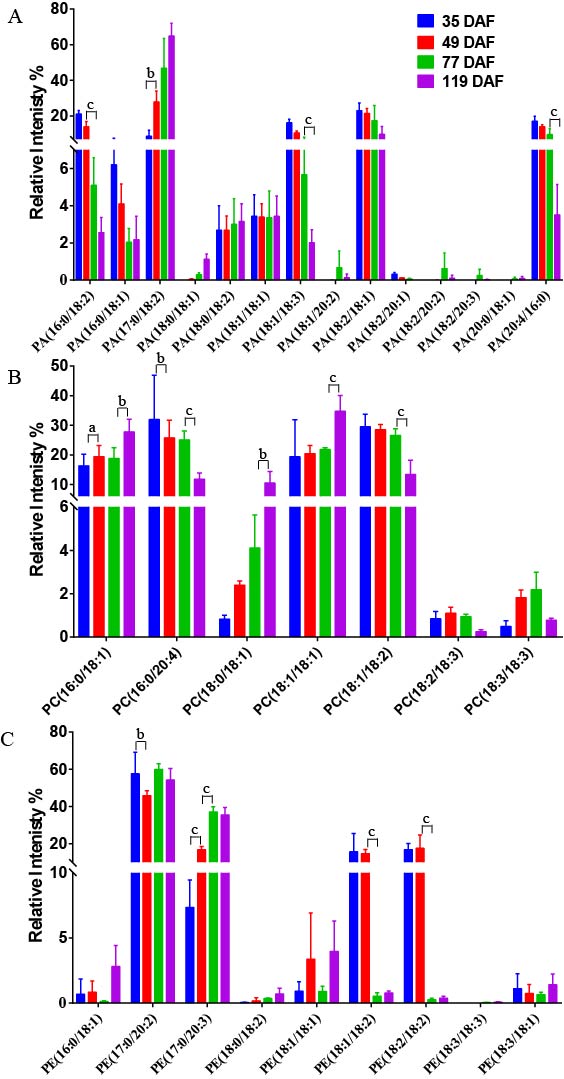


Supplementary Figure S5. Changes in the percentage of major molecular species of glycerol phospholipids during development of *Paeonia ostii* kernel.

A, molecular species of PA; B, molecular species of PC; C, molecular species of PE. Means±s.d. (n=8) were shown. a p<0.05, b p<0.01, c p<0.001. Major molecular species (> 0.5% total) were detailed here.


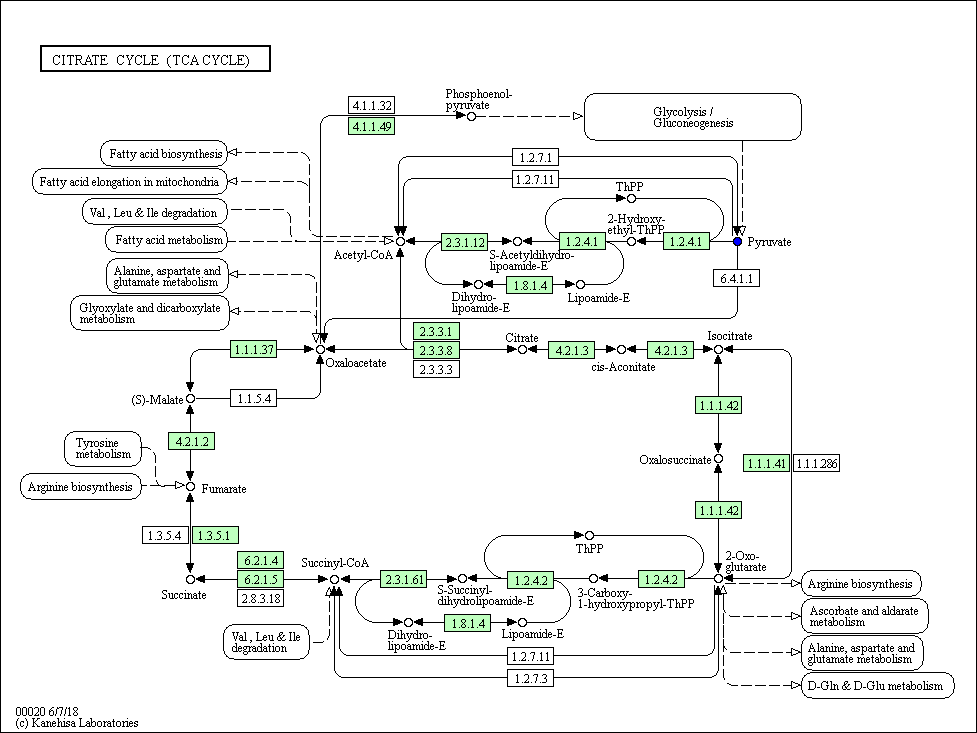


Supplementary Figure S6. Citrate cycle (TCA cycle) pathway with significant enrichment.

The blue circle indicates the decreased pyruvic acid. This image is obtained by KEGG, and the map number is 00020.


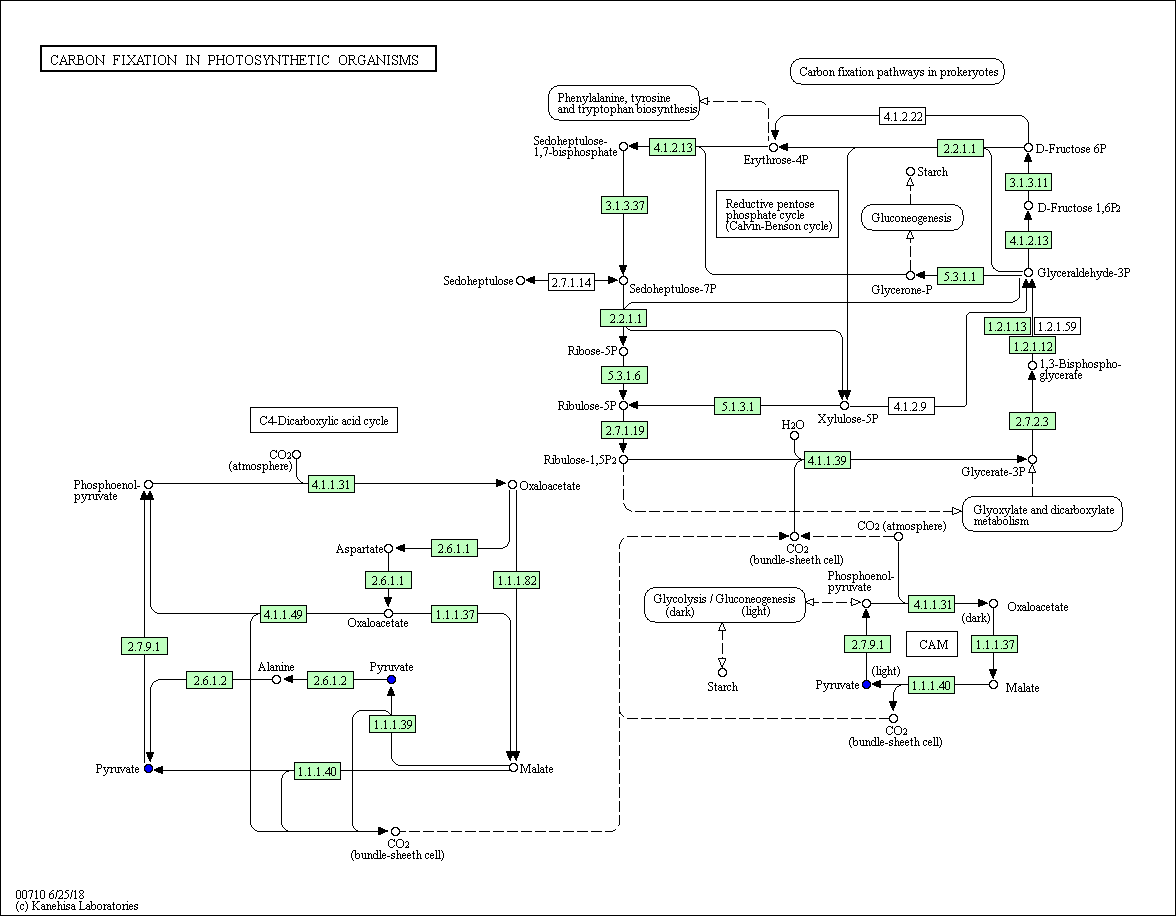


Supplementary Figure S7. Carbon fixation in photosynthetic organisms pathway with significant enrichment.

The blue circle indicates the decreased pyruvic acid. This image is obtained by KEGG, and the map number is 00710.


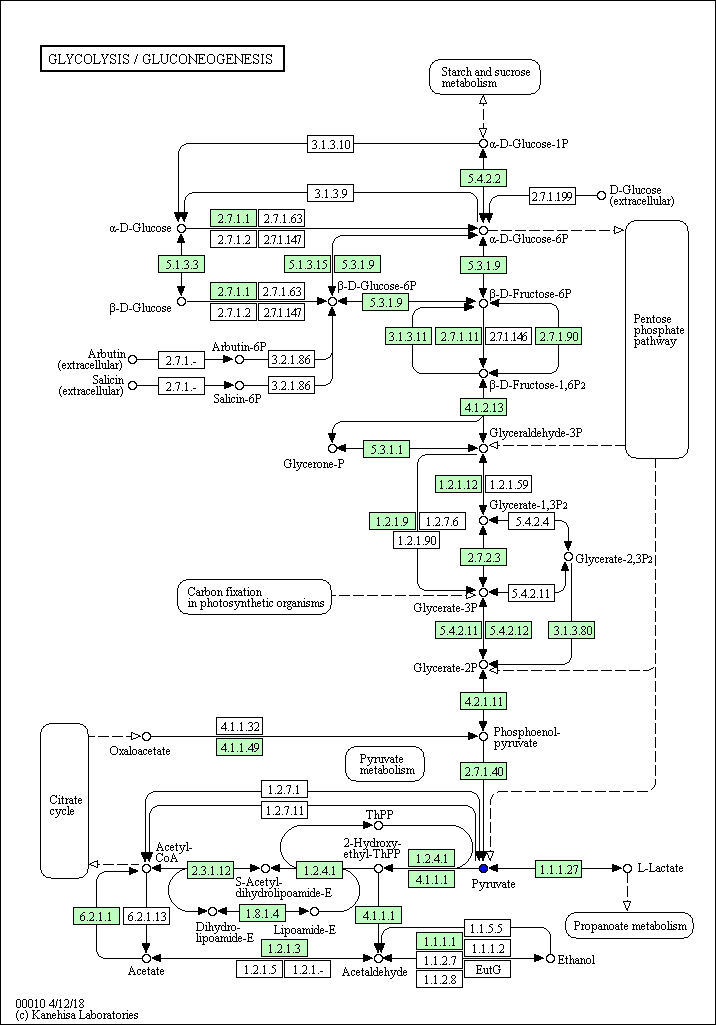


Supplementary Figure S8. Glycolysis/Gluconeogenesis pathway with significant enrichment.

The blue circle indicates the decreased pyruvic acid. This image is obtained by KEGG, and the map number is 00010.

Supplementary Table S2 Molecular species of phospholipids identified in the *Paeonia ostii* kernel.

| Lipid name (C:N) | m/z | t_R_(min) | Adducts | Acyl chain composition |
| --- | --- | --- | --- | --- |
| PA (32:1) | 664.4909 | 6.2235 | M+NH4 | 14:0-18:1 |
| PA (32:2) | 662.4753 | 5.1662 | M+NH4 | 18:2-14:0 |
| PA (33:0) | 661.4832 | 7.8721 | M-H | 17:0-16:0 |
| PA (33:1) | 659.4675 | 6.2494 | M-H | 15:0-18:1 |
| PA (33:2) | 681.4455 | 5.2305 | M+Na | 13:0-20:2 |
| PA (34:1) | 692.5214 | 7.9963 | M+NH4 | 16:0-18:1 |
| PA (34:2) | 690.5058 | 6.5963 | M+H | 16:0-18:2 |
| PA (34:2) | 695.46209 | 6.1958 | M+Na | 14:0-20:0 |
| PA (34:2) | 695.4629 | 3.1509 | M+Na | 18:1-16:1 |
| PA (34:3) | 688.4892 | 3.5219 | M+NH4 | 18:3-16:0 |
| PA (35:2) | 1395.9698 | 6.5963 | 2M+Na | 15:0-20:2 |
| PA (35:2) | 704.5213 | 6.5963 | M+Na | 17:0-18:2 |
| PA (35:3) | 702.5060 | 3.5980 | M+H | 17:0-18:3 |
| PA (36:1) | 720.5534 | 9.1801 | M+NH4 | 18:0-18:1 |
| PA (36:2) | 699.5001 | 8.0843 | M-H | 18:0-18:2 |
| PA (36:2) | 718.5371 | 8.1305 | M+NH4 | 18:1-18:1 |
| PA (36:3) | 716.5217 | 4.3122 | M+NH4 | 18:0-18:3 |
| PA (36:3) | 716.5214 | 6.6348 | M+NH4 | 18:2-18:1 |
| PA (36:4) | 695.4687 | 5.4696 | M-H | 18:1-18:3 |
| PA (36:4) | 714.5055 | 3.5790 | M+NH4 | 18:3-18:1 |
| PA (36:4) | 714.5061 | 5.4779 | M+NH4 | 20:4-16:0 |
| PA (36:6) | 710.4771 | 1.143 | M+H | 18:3-18:3 |
| PA (38:1) | 729.5466 | 11.0051 | M-H | 20:0-18:1 |
| PA (38:2) | 727.5310 | 8.5653 | M-H | 20:1-18:1 |
| PA (38:3) | 725.5153 | 6.9884 | M-H | 18:1-20:2 |
| PA (38:3) | 744.5531 | 8.4129 | M+NH4 | 18:2-20:1 |
| PA (38:4) | 723.4998 | 5.8944 | M-H | 18:2-20:2 |
| PA (38:5) | 721.4843 | 4.9317 | M-H | 18:2-20:3 |
| PA (39:1) | 762.5984 | 11.8491 | M+NH4 | 18:1-21:0 |
| PA (39:3) | 758.5684 | 9.1221 | M+NH4 | 18:3-21:0 |
| PA (41:3) | 786.5994 | 11.5608 | M+NH4 | 21:0-20:3 |
| PC (33:2) | 742.5406 | 6.7959 | M-H | 15:0-18:2 |
| PC (33:2) | 742.5426 | 5.8368 | M-H | 15:1-18:1 |
| PC (34:1) | 760.5835 | 8.3423 | M+H | 16:0-18:1 |
| PC (36:1) | 788.6149 | 10.9110 | M+H | 18:0-18:1 |
| PC (36:2) | 786.5996 | 8.4769 | M+H | 18:1-18:1 |
| PC (36:3) | 784.5839 | 6.8852 | M+H | 18:1-18:3 |
| PC (36:4) | 782.5680 | 5.8046 | M+H | 16:0-20:4 |
| PC (36:5) | 824.5481 | 4.8544 | M+FA-H | 18:2-18:3 |
| PC (36:6) | 778.5370 | 4.1973 | M+H | 18:3-18:3 |
| PC (38:1) | 816.6456 | 11.9249 | M+H | 18:1-20:0 |
| PC (38:3) | 812.6154 | 8.7623 | M+H | 18:2-20:1 |
| PE (34:1) | 716.5267 | 8.908 | M-H | 16:0-18:1 |
| PE (34:2) | 738.5061 | 5.1163 | M+Na | 16:0-18:2 |
| PE (36:2) | 744.5536 | 9.3918 | M+H | 18:0-18:2 |
| PE (36:2) | 744.5537 | 9.0713 | M+H | 18:1-18:1 |
| PE (36:3) | 740.5261 | 4.9126 | M-H | 18:1-18:2 |
| PE (36:4) | 738.5107 | 4.2117 | M-H | 18:2-18:2 |
| PE (36:4) | 740.5218 | 6.1607 | M+H | 18:3-18:1 |
| PE (36:6) | 736.4908 | 4.3838 | M+H | 18:2-18:3 |
| PE (37:2) | 802.5641 | 6.7959 | M+FA-H | 17:0-20:2 |
| PE (37:3) | 800.5483 | 5.7208 | M+FA-H | 17:0-20:3 |
| PG (33:2) | 713.4793 | 3.5682 | M-H2O-H | 15:0-18:2 |
| PG (34:1(O)) | 715.5314 | 10.4293 | M-H2O-H | 16:0-18:1 |
| PG (34:2(O)) | 713.5156 | 8.4112 | M-H2O-H | 16:0-18:2 |
| PG (34:2) | 745.5057 | 6.009 | M-H | 18:2-16:0 |
| PG (34:3) | 743.4897 | 5.1315 | M-H | 18:3-16:0 |
| PI (34:1) | 835.5373 | 6.8923 | M-H | 16:0-18:1 |
| PI (34:3) | 850.5424 | 4.9135 | M+NH4 | 18:2-16:1 |
| PI (34:3) | 831.5061 | 4.8739 | M-H | 18:3-16:0 |
| PI (36:5) | 874.5428 | 4.2358 | M+NH4 | 18:2-18:3 |
| PS (36:1) | 818.5587 | 3.6452 | M+FA-H | 16:0-20:1 |
| PS (38:0) | 818.5956 | 9.5446 | M-H | 17:0-21:0 |
| PS (44:3) | 920.6387 | 11.6954 | M+Na | 22:2-22:21 |

The identification of the selected lipid compounds is mainly based on the accurate mass number, secondary fragments and isotope distribution. Considering three factors, the full score of each item is 20 points, and the total score of the score is 60 points. Among them, the mass spectrum information of the secondary fragments is relatively important, and the full score is 100 points as the fragmentation score item. According to the score of fragmentation score higher than 60 and score higher than 45, some TAGs molecular species were selected. C:N represents the total number of carbons (C) and the number of double bonds (N). t_R_, retention time.
